# Illinois State Medical Society

**Published:** 1869-06-01

**Authors:** 


### *Illinois State Medical Society.*

[We are indebted to the enterprising reporters of the *Chicago Times* for the following *résumé*.—Eds.]

### MORNING SESSION.

The Illinois State Medical Society opened its Ninteenth annual session on May 18th, at 10 o'clock, in the Common Council chamber.

### CALLED TO ORDER.

The Convention was called to order by Dr. S. T. Trowbridge, the president of the association, and the following delegates were reported as being present:

### LIST OF DELEGATES.

Drs. S. T. Trowbridge, of Decatur; J. N. Burns, of Decatur; S. W. Noble, of Bloomington; H. Noble, of Heyworth, McLean county; P. R. Pope, of Bloomington; J. T. Wilson, of Quincy; R. C. Hamill, of Chicago; N. S. Davis, of Chicago; B. F. Barnes, of Taylorville, Christian county; J. P. Matthews, of Carlinville; A. Niles, of Quincy; H. B. Buck, of Springfield; James H. Reynolds, of Quincy; J. F. McCormick, of Fowler Quincy Medical College; S. W Albin, of Neoga, Cumberland county; E. Ingalls, of Chicago; G. C. Paoli, of Chicago; T. J. Fitch, of Chicago; J. C. Corbines, of Mendota; W. A. Knox, of Chicago; J. M. Hutchinson, of Chicago; E. P. Cook, of Mendota; W. A. Knox, of Chicago; T. B. Hallen, of Vandalia; J. O. Hamilton, of Jerseyville, Jersey county; Edwin Powell, of Chicago Rush Medical College; L. H. Baker, of Quincy; S. A. McWilliams, of Chicago; W. R. Fox, of Wilmington; A. Fisher, of Chicago; D. L. Jewitt, of Watseka; Elias Wanzer, of Gillman, Iroquois county; R. G. Bogue, of Chicago, Cook County Hospital; E. L. Holmes, of Chicago Eye and Ear Infirmary; J. T. Penman, of Champaign; De Laskie Milier, of Chicago; P. S. McDonald, of Chicago; H. M. Hunt, of Chicago; J. S. Hildreth, of Chicago;

Swayne Wickersham, of Chicago; Curtis T. Fenn, of Chicago; Moses Gunn, of Chicago; J. H. Hollister, of Chicago.

SECRETARY.

Dr. N. S. Davis, of Chicago, secretary of the convention.

PERMANENT MEMBERS.

On motion, the following gentlemen were elected permanent members of the society:

Drs. G. W. Bane, of Macomb; D. M. Vosburgh, of Earlville, La Salle county; W. M. Burbank, of Bloomingdale county; E. R. Willard, of Wilmington; C. A. W. Zimmerman, of Quincy; Chas. Rutler, of Chicago; W. C. Sequert, of Watseka; John M. Woodworth.

ADDRESS OF WELCOME.

Dr. Hamill, chairman of the committee of arrangements, opened the convention by a brief address of welcome to the delegates. He spoke of the important and arduous duties of the medical profession, and of the necessity of medical men meeting together for the purpose of discussion. These annual meetings of the society were proving to be not only of great interest but of great value. It was to be hoped that much good would be achieved by the deliberations of the present session.

In conclusion, the gentleman tendered to the delegates invitations to visit the several hospitals of the city, the Academy of Sciences, and Historical society.

HOURS FOR MEETING.

The hours of meeting, as determined upon by committee, were as follows: Opening day—morning session, 10 A. M. to 1 P. M.; afternoon session, 3 P. M., to 6 P. M.

Other Days—Morning session, 9 A. M., to 1 P. M..; afternoon session, 2½ P. M., to 6 P. M.

TREASURER.

Dr. E. L. Holmes, of Chicago, was, on motion, elected treasurer *pro tem.* of the convention.

A VISITOR FROM NEW YORK.

Dr. Hiram Corliss, of Washington county, New York, a delegate from the New York Medical society to this convention, was formally presented to the delegates, and in response spoke briefly of the history and condition of the medical society represented by him.

OFFICERS.

The election of officers, which was next in order, was, on motion of Dr. N. S. Davis, of Chicago, temporarily postponed.

LETTERS

from Dr. Wright, of Chatham, and other gentlemen, unable to be present at the convention, were read.

PRACTICAL MEDICINE AND EPIDEMICS.

A report was then read from the committee on practical medicine and epidemics by Dr. E. P. Cook, of Mendota, the chairman. The committee premise their report with the statement that, in their search for information they had addressed printed circulars to physicians in every county in the state, but had only received three or four answers. For this reason the report of the committee would not be as full as might be desired. The report considered that in the large cities of the state, sufficient attention is perhaps devoted to the sanitary laws, though in almost all the communities there exists a necessity for a more accurate registration of births, deaths, and marriages. One fact of interest to the profession during the year was a marked change in the nature of the diseases from those that have prevailed during the same time for many years. In regard to the climatology, it was stated that the year had opened with an extended drought which, toward the middle, was followed by heavy rains, which in turn gave place to a season of very hot weather. During this heated term, cases of *coup de soleil* had been not unfrequent. In this regard the committee regretted the fact that the nature of and best immediate remedies for this class of the causes of death had not received the attention of the profession that their importance demanded. Even the best authorities were divided in opinion upon the subject. The committee recommended the theme to the serious consideration of the convention.

Another point noticed in the report was the prevalence of malarious fevers, during the months of September and November. In the neighborhood of Princeton, a species of this class of fevers, described as typho-malarial, had been noticed in a marked form, and in the case of a large number of sufferers. The committee spoke at length of the diagnosis of the disease, as contrasted with ordinary typhoid and scorbutic fevers, and the best medical agents to be used.

The report, which closed with much valuable information, was then, on motion, accepted, and referred to the committee on publication.

### ANNUAL ASSESSMENTS.

On motion, the annual assessments of the society were continued at $3.

### TREASURER'S REPORT.

Dr. J. H. Hollister, the treasurer of the society, submitted his annual report, as follows:

| | |
|---|---|
| Balance on hand, May, 1868 | $ 43 39 |
| Receipts from last annual | |
| Meeting at Quincy | 146 00 |
| Other receipts | 45 00 |
| Total | $234 39 |
| Expenditures | 172 68 |
| Balance on hand | $ 61 71 |

The report was accepted and referred to the auditing committee.

### EPIDEMICS.

A supplemental report upon the subject of practical medicine and epidemics was then read by Dr. D. L. Jewell, of Watseka, of the committee. The gentleman confined his report chiefly to a sketch of the extent and nature of scarlet fever epidemic, which had characterized the past year, in the eastern portion of Iroquois county. The symptoms of several individual patients were described and commented upon, and the remedies employed stated. Dr. Jewell, however, read his paper so indistinctly that it was very difficult for any one not in his immediate vicinity to hear the nature of his remarks. The epidemic he noticed in Iroquois county commenced in the month of February, 1868, and rapidly spread throughout the district.

The report was, on motion, received and referred to the committee on publication.

### A VISIT TO THE HOSPITALS.

On motion, the convention resolved to visit the several medical colleges and hospitals, from which invitations had been received, as a society, the time of the visits to be determined upon by the committee of arrangements.

### AN INVITATION.

An invitation was extended to the delegates, by Dr. N. S. Davis, to visit him, with their wives, at his residence on Wabash avenue, this evening, at 8 o'clock.

### AUDITING COMMITTEE.

On motion, each county represented in the convention, was authorized to select one member, for the purpose of forming a committee for the nomination of officers.

The society then adjourned until 3 o'clock in the afternoon.

### AFTERNOON SESSION.

The second session of the society was commenced at 3 o'clock in the afternoon, pursuant to adjournment, the president in the chair.

### PERMANENT MEMBERS.

On motion, Dr. S. J. Jones, of Chicago; Dr. Peter Eppler, of Cayuga; Dr. D. B. Trimble, of Chicago; Dr. John B. Hamilton, of Jerseyville, Dr. R. D. Bradley, of Bloomington and Dr. John Rauch, of Chicago, were elected permanent members of the association.

### OBSTETRIES.

Dr. H. B. Buck, Springfield, chairman of the committee on obstetrics, read an extensive and, to the profession, highly interesting report. The gentleman strongly urged the necessity of not only considerable professional knowlege, but long practical experience on the part of the practitioner, in order to allow him to practice this important branch of the profession with safety and success. He warmly advocated the system of keeping record of all cases in obstetrics, attended by the physician, inasmuch as the testimony of a number of witnesses would tend much to clear away many existing difficulties. In the course of his report, the speaker cited many cases of professional interest which had come beneath the experience of himself, and other physicians with whom he had been associated. A delegate moved that the report be accepted, and referred to the publication committee.

The motion, after a few remarks by Dr. Corliss, of New York, narrating certain difficult obstetrical cases attended by him, was carried.

### NOMINATING COMMITTEE.

The committee on nomination of officers was appointed, as follows:

Cumberland county, G. W. Albin; Adams county, A. Miles; Cook county, R. G. Bogue; McLean county, H. Noble; Will county, W. R. Knox; Fayette county, F. B. Haller; Champaign county, G. T. Penman; La Salle county, J. C. Corbus; Christian county, B. F. Barnes; Macoupin county, J. P. Mathews; McDonough county, G. H. Bane; De Kalb county, H. F. Page; Sangamon county, H. B. Buck; Macon county, I. N. Barns; Iroquois county, Elias Wanzer; Jersey county, J. Hamilton; Livingston county, P. Eppler.

### RESIGNATION.

The resignation of Dr. N. S. Davis as permanent secretary of the society, after having held the position for 12 years, was tendered.

The resignation was, on motion, accepted, and a vote of thanks tendered to the gentleman for the able and faithful manner in which he has discharged the duties of his office.

### APHASIA.

Dr. Hurd, of Galesburg, appointed, at the last meeting, a committee to report a paper on aphasia, not having completed the article, was requested to perfect it as soon as possible, and submit it to the publication committee.

### CHOLERA INFANTUM.

Dr. N. Wright, of Chatham, and H. C. Barrell, of Springfield, were continued until next year as a committee on cholera infantum.

### SURGICAL APPARATUS.

On motion of Dr. Davis, of Chicago, the order for the first hour of this morning's session, was determined to be the exhibition of surgical apparatus for treating fractures of the leg, femur, head of humerus, olecranon process, and lower jaw, by Dr. E. A. Clark, of St. Louis.

### STAPHYLORAPHY.

Dr. Moses Gunn, of Chicago, the committee on staphyloraphy, then read a report upon the subject, premising his remarks by the regret that no extended information could be obtained upon the subject. It was first practiced, though unsuccessfully, by a German physician in 1817. Two years later, another European practitioner, though unaware of the previous operation in Germany, performed it successfully. A little later, the elder Warren of Boston, also ignorant of the above named efforts, was successful in the same operation. All these cases, however, simply involved the closure of the partial fissures in the palate, and it was not until some years later, that Dr. J. Mason Warren, a son of the above named surgeon, planned a successful operation for the closure of a complete fissure in the arch. In continuing his report, the gentleman cited many cases reported to him by some of the leading surgeons of the country, and three that had come under his own experience; though in the latter, and in many of the former cases, no improvement to articulation was achieved.

Dr. Corliss, of New York state, cited the case of an operation performed by Dr. Buck, of New York, in which, after the operation, the patient was afforded the means of speech by an artificial palate made by a dentist. Dr. Buck, the speaker considered, was probably the most successful exponent of plastic surgery in the world.

Dr. N. S. Davis, of Chicago, did not consider that in this case the improvement to speech was due to the surgical operation of Dr. Buck, so much as to the artificial instrument supplied by the dentist.

The report of Dr. Gunn was then referred to the committee on publication.

### REMOVAL OF CATARACT.

A volunteer report upon the methods of increasing success in the removal of cataracts from the eye was read by Dr. J. S. Hildreth, of Chicago, and referred to the committee on publication.

### ADDITIONAL DELEGATES.

The following additional delegate to the convention were reported:

Drs. D. O Crist, of Bloomington; H. W. Boyd, of Chicago; M. O. Heydock, of Detroit Medical college; N. T. Quales, of Chicago: A. Grosbeck, of Chicago; H. P. Merriman, of Chicago; R. N. Isham, of Chicago; G. R. Russ, Cook County hospital; D. T. Nelson, of Chicago; J. V. Z. Blaney, of Chicago (permanent member); H. F. Page, of Sycamore; W. H. Byford, of Chicago; E. Holderness, of

Tonawanda; Thomas Bevan, of Chicago; S. J. Avery, of Chicago; E. O. F. Roler, of Chicago (permanent member); V. L. Hurlburt, of Chicago; J. Adams Allen, (permanent member.)

THE NEXT MEETING.

A communication was received from the Bloomington Medical society, offering accommodations for the next meeting of the society in that city.

The society then adjourned until 9 o'clock this morning.

SECOND DAY—MORNING SESSION.

The Illinois Medical society commenced the second day's session of its annual meeting, May 19th, at 8 o'clock, pursuant to adjournment, the president in the chair.

According to the resolution of the previous afternoon, the first hours of the session were devoted to the exhibition by Dr. Clarke, of St. Louis, of surgical apparatus of his own invention, for the treatment of simple and compound fractures of the leg.

The apparatus of Dr. Clarke was explained in detail, and appeared to give general satisfaction to all the gentlemen present.

In reply to the question from Dr. Corliss, of New York, Dr. Clarke said that he did not believe in the value of starch bandages in cases of compound fractures of the leg unless the fracture be oblique, and there is no shortening.

NEW MEMBERS.

Dr. J. Van Dyke, of Oswego, N. Y., was elected a member of the society by invitation, and the following gentlemen were also elected members of the association: Drs. W. C. Lyman, of Chicago; Samuel J. Jones, of Chicago; Nelson Lowrin, of Chicago; E. Marguerat, of Chicago; Charles Hunt, of Dixon, and J. R. Corbus, of Amboy,

ADDITIONAL DELEGATES.

The following additional delegates were announced as being present: Drs. C. W. Oleson, Bloomingdale; A. Ward, of Aurora; R. J. Patterson, of Batavia; D. S.

Jenks, of Plano; S. P. Bread, of Bureau, D. L. Ware, of Chicago.

NOMINATION OF OFFICERS.

The committee on nominations, through its chairman, Dr. H. Noble, reported the following ticket for election for the ensuing year,

President—Prof. J. V. Z. Blaney, of Chicago.

First Vice President—Dr. G. W. Albin, of Neoga.

Second Vice President—Dr. H. B. Buck, of Springfield.

Treasurer—Dr. J. H. Hollister, of Chicago.

Permanent Secretary—Dr. T. L. Fitch, of Chicago.

Assistant Secretary—Dr. Chas. Hunt, of Dixon.

Place of meeting next year—Bloomington, Ill.

COMMITTEES.

The following committees were also nominated:

Practice of Medicine—Dr. C. Goodbrake, of Clinton; Dr. J. S. Whitmire, of Metamora; Dr. Ira Barnes of Decatur.

Surgery—Dr. oses Gunn, of Chi cago; Dr. G. R. Bibb, of Jacksonville: Dr. John B. Hamilton, of Jerseyville.

Obstetrics—Dr. Elias Wanzer, of Gillman; Dr. J. T. Penman, of Champaign; Dr. D. R. Breed, of Princeton.

Drugs and Medicines—Dr. Charles Hunt, of Dixon; Dr. C. R. Park, of Bloomington; Dr. T. G. Hickman, of Vandalia.

Necrology—Dr. J. H. Hollister, of Chicago; Dr. F. B. Haller, of Vandalia; Dr. J. O. Hamilton, of Jerseyville.

SPECIAL COMMITTEES.

The following special committies were also nominated:

Criminal Abortions—Dr. De Laskie Miller, of Chicago; Dr. W. H. Byford, of Chicago; Dr. H. Noble, of Bloomington.

Opthalmology—Drs. J. S. Hildreth, E. L. Holmes, and S. J. Jones, of Chicago.

Pulmonary Tuberculosis—Dr. J. R. Ross, of Chicago; J J. N. Viglas, of Peoria; T. D. Washburn, of Hillsboro.

Use of Plaster of Paris in Fractures—Dr. R. G. Bogue, of Chicago.

Otology—Drs. Samuel J. Jones, of Chicago; J. R. Corbus, of Amboy; and J. S. Hildreth, of Chicago.

Committee on Arrangements—Drs. Everett, Phillips, Hunt, J. C. Corbus, and E. P. Cook, of Dixon.

PLACE OF MEETING.

The report was so amended as to fix the place for the next annual meeting at Dixon, instead of Bloomington, and as so amended was unanimously adopted, and ordered published.

LITHOTOMY.

An able and valuable paper upon lithotomy was reported by Dr. E. Powell, of Chicago, and after some discussion upon points brought out by the reports, was accepted and referred to the committee on publication.

INVITATIONS.

Dr. S. J. Jones, of this city, extended an invitation to the society to visit his office, No. 22 Washington street, this evening, for the purpose of inspecting some surgical instruments which had been obtained by him from Europe.

The invitation of Dr. Davis for the delegates, with their ladies, to visit him at his residence, No. 797 Wabash avenue, in the evening, was again read for the benefit of those delegates who had arrived during the morning.

DRUGS AND MEDICINES.

Dr. N. S. Davis, of Chicago, chairman of the committee on drugs and medicines, read the report of that committee upon the subject. The report was of an able and explanatory character, embracing the improvements that had been effected during the year, in the introduction of new remedial agents, and also to notice the practice of the adulteration of drugs.

After a brief discussion regarding the relative merits of peroxide of hydrogen, mentioned in the report as a powerful agent in adding oxygen to the blood, and thereby imparting vitality to the system; and the permanganate of potash, which has been used to produce the same effect, with marked success by several gentlemen present, the convention adjourned until afternoon.

IN THE AFTERNOON

the delegates visited the several hospitals and medical colleges in the city, and

IN THE EVENING

accepted the invitation of Dr. N. S. Davis, and visited him with their ladies, at his residence on Wabash avenue. Here a very pleasant time was spent and the party broke up at a reasonable hour, highly pleased with the hospitality of their entertainer.

LAST DAY'S SESSION THURSDAY, MAY 20, 1869.

The Illinois State Medical society re-assembled in the council chamber of the court house, Dr. S. F. Trowbridge, president of the society, in the chair.

REMEDIES.

Dr. D. Ingalls read a paper on "Remedies," which was principally a plea in favor of assisting rather than inducing nature to the work of restoration. In this connection he strongly denounced the exploded barbarities which obtained with old-time surgeons, and deprecated the use of sanguinary measures in the treatment of disease as unwarranted by the present condition of science. The prejudice cherished in almost every family, in favor of simple but strong cathartics, was also reproved. He preferred to aid the organs of the human system, in their efforts to repair the ravages of disease, rather than to ignore this subtle process, and to interfere violently with instruments or drugs. The address was very well received by the meeting, and was referred to the committee of publication.

NECROLOGY.

Dr. J. H. Hollister, of the committee on necrology, presented his mortuary report, from which it appeared that only one of the members of the original society, organized nineteen years ago in Springfield, was alive. The doctor then paid brief tributes to the memories of several of the illustrious dead, commencing with the obituary of Prof. B. W. Herrick, first president of the society.

The report was referred to the committee on publication.

ANÆSTHETICS.

Dr. E. Andrews presented a report on the comparative merits of the various anæsthetics known to the professsion. He declared that nitrous oxide gas was the safest and pleasantest of all, but that its effects were too temporary to be useful in a protracted surgical operation.

An animated debate followed, in which Drs. Gunn, Fitch, Hildreth, Andrews, Ingalls and Hollister participated, each submitting cases which had come under his cognizance.

INSANITY.

Dr. R. J. Patterson, of Batavia, who had prepared a paper on the legal conditions of insanity, having left by an early train, his essay was read by the Secretary. It was a dissertation upon the legal points involved in every case of lunacy, and the state laws with reference to lunatic asylums. The paper was referred to the committee on publication.

A VOTE OF THANKS.

A vote of thanks was then tendered to the common council, the city physicians, and the officers of the society.

ADJOURNMENT.

The meeting then adjourned *sine die*.

